# ACTL6A suppresses p21^Cip1^ tumor suppressor expression to maintain an aggressive mesothelioma cancer cell phenotype

**DOI:** 10.1038/s41389-021-00362-7

**Published:** 2021-10-23

**Authors:** Suruchi Shrestha, Gautam Adhikary, Warren Naselsky, Wen Xu, Joseph S. Friedberg, Richard L. Eckert

**Affiliations:** 1 Department of Biochemistry and Molecular Biology, University of Maryland School of Medicine, Baltimore, MD USA; 2 Department of Surgery, University of Maryland School of Medicine, Baltimore, MD USA; 3 Marlene and Stewart Greenebaum Comprehensive Cancer Center, University of Maryland School of Medicine, Baltimore, MD USA; 4 Department of Dermatology, University of Maryland School of Medicine, Baltimore, MD USA; 5 Department of Reproductive Biology, University of Maryland School of Medicine, Baltimore, MD USA

**Keywords:** Cancer, Molecular biology

## Abstract

Mesothelioma is a poor prognosis cancer of the mesothelial lining that develops in response to exposure to various agents including asbestos. Actin-Like Protein 6A (ACTL6A, BAF53a) is a SWI/SNF regulatory complex protein that is elevated in cancer cells and has been implicated as a driver of cancer cell survival and tumor formation. In the present study, we show that ACTL6A drives mesothelioma cancer cell proliferation, spheroid formation, invasion, and migration, and that these activities are markedly attenuated by ACTL6A knockdown. ACTL6A expression reduces the levels of the p21^Cip1^ cyclin-dependent kinase inhibitor and tumor suppressor protein. DNA binding studies show that ACTL6A interacts with Sp1 and p53 binding DNA response elements in the p21^Cip1^ gene promoter and that this is associated with reduced p21^Cip1^ promoter activity and p21^Cip1^ mRNA and protein levels. Moreover, ACTL6A suppression of p21^Cip1^ expression is required for maintenance of the aggressive mesothelioma cancer cell phenotype suggesting that p21^Cip1^ is a mediator of ACTL6A action. p53, a known inducer of p21^Cip1^ expression, is involved ACTL6A in regulation of p21^Cip1^ in some but not all mesothelioma cells. In addition, ACTL6A knockout markedly reduces tumor formation and this is associated with elevated tumor levels of p21^Cip1^. These findings suggest that ACTL6A suppresses p21^Cip1^ promoter activity to reduce p21^Cip1^ protein as a mechanism to maintain the aggressive mesothelioma cell phenotype.

## Introduction

Mesothelioma is an aggressive asbestos-triggered cancer arising from the mesothelial layer of the pleura and peritoneum that is considered incurable and is associated with poor life expectancy [1, 2]. Surgical resection and chemotherapy are standard of care and drug resistance and cancer recurrence is a common problem [2, 3]. Our goal is to identify proteins that promote and maintain the aggressive mesothelioma cancer cell phenotype as potential therapy targets [4, 5].

SWI/SNF is a large multi-subunit epigenetic regulatory complex that acts as a tumor suppressor by controlling nucleosome spacing, chromatin structure, and transcription [6, 7]. However, some components of this complex have independent functions. An important example is Actin-Like Protein 6A (ACTL6A), which is part of the SWI/SNF complex [8], but also acts independently to drive cancer cell survival [9–12]. As an independent regulator, ACTL6A maintains stem cell self-renewal [13], acts as a c-myc cofactor to drive oncogenesis [14] and regulates epithelial–mesenchymal transition [15–17].

In addition, ACTL6A stabilizes the YAP1/TAZ pro-cancer transcriptional regulators, which are part of the Hippo signaling cascade [18, 19], and reduces expression of the p21^Cip1^ tumor suppressor [20–23]. We are interested in the role of ACTL6A in mesothelioma, as YAP1/TAZ function is required for optimal mesothelioma cancer cell survival [24]. Moreover, expression of the p21^Cip1^ cyclin-dependent kinase inhibitor is induced by various anti-cancer agents and p21^Cip1^ expression is associated with reduced mesothelioma cancer cell survival [25–27]. Our present study suggests that ACTL6A maintains the aggressive mesothelioma cancer phenotype by interacting with the p21^Cip1^ gene promoter to inhibit transcription and reduce p21^Cip1^ expression. Moreover, elevated p21^Cip1^ is required to suppress the cancer phenotype, as the phenotype persists when the p21^Cip1^ increase is inhibited in ACTL6A deficient cells. Our findings support a model where ACTL6A interacts with the Sp1 and p53 response elements in the p21^Cip1^ promoter to reduce p21^Cip1^ expression, and that loss of p21^Cip1^ tumor suppressor enhances the mesothelioma cancer phenotype.

## Results

### ACTL6A maintains the aggressive mesothelioma cancer phenotype

**Fig. 1:**
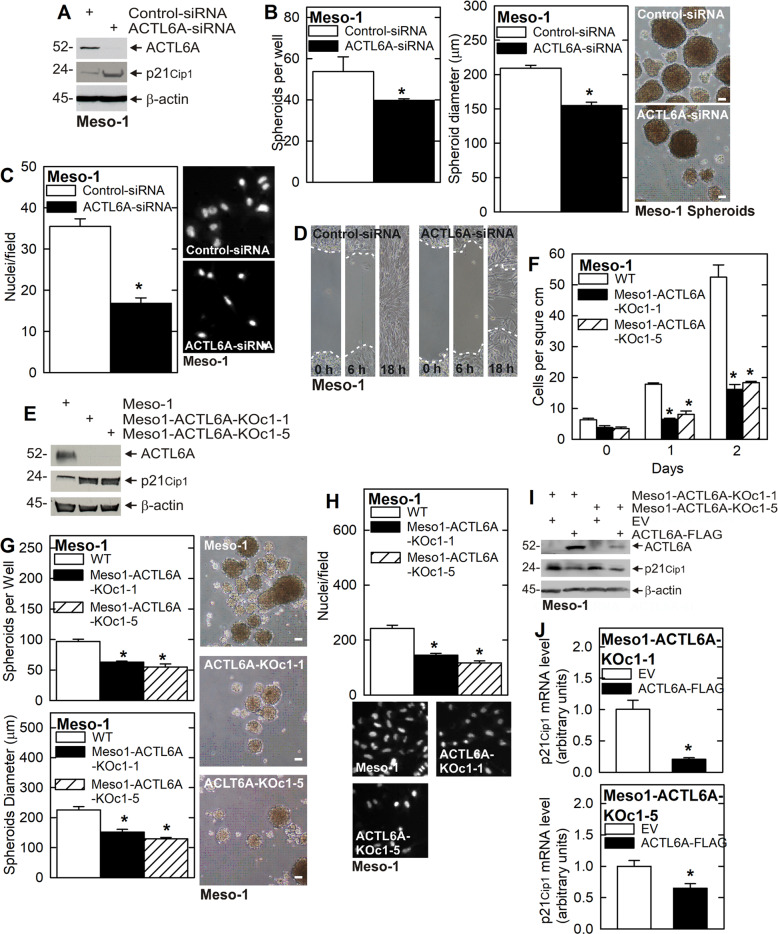
ACTL6A maintains the aggressive cancer phenotype. **A** ACTL6A was successfully knocked down using ACTL6A-siRNA which leads to an increase in p21^Cip1^ protein level. **B**–**D** ACTL6A knockdown decreases spheroid number and size, invasion and migration. For spheroid assay, 40,000 cells were seeded on ultra-low attachment plates and counted at Day 4. Spheroid diameters were measured using ImageJ. **E**–**H** ACTL6A knockout cell clones, which lack ACTL6A, display elevated p21^Cip1^ expression and this is associated with reduced cell proliferation, spheroid formation and invasion. **I**, **J** ACTL6A overexpression in ACTL6A knockout cell clones reduces p21^Cip1^ protein and mRNA levels. Asterisks indicate a significant decrease (*n* = 3, *p* ≤ 0.001).

ACTL6A has been reported to maintain an aggressive phenotype in cancer cells [9–12]. Enhanced spheroid formation, invasion through Matrigel and migration on plastic are properties associated with the aggressive mesothelioma cancer phenotype [4] and so we measured the impact of ACTL6A on these properties. As shown in Fig. 1A, ACTL6A knockdown is associated with increased levels of p21^Cip1^ and this is associated with reduced spheroid size and number, invasion and migration (Fig. 1B–D). To further characterize the role of ACTL6A, we characterized ACTL6A knockout cells. The ACTL6A-KOc1-1 and ACTL6A-KOc1-5 clonal cell lines display a marked increase in p21^Cip1^ expression (Fig. 1E) and this is associated with reduced cell proliferation, spheroid number and diameter, and cell invasion (Fig. 1F–H). We next performed the inverse experiment and restored ACTL6A expression in ACTL6A knockout cells. We show that restoring ACTL6A level is associated with reduced p21^Cip1^ mRNA and protein expression (Fig. 1I, J) suggesting that ACTL6A controls p21^Cip1^ level by regulating p21^Cip1^ RNA turnover or gene transcription.

### p21^Cip1^ suppresses the peritoneal and pleural mesothelioma cancer cell phenotype

**Fig. 2:**
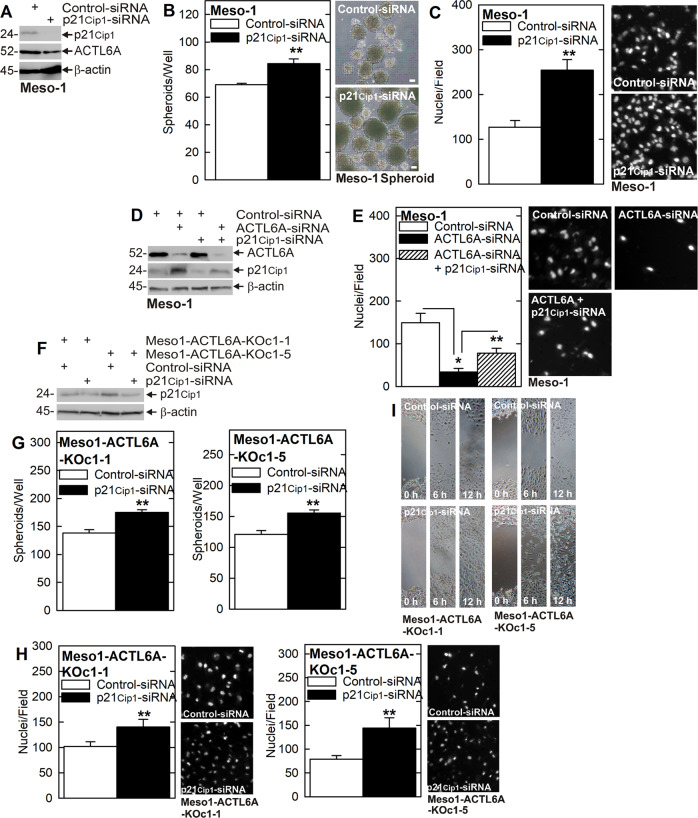
p21^Cip1^ suppresses the cancer phenotype. **A**–**C** p21^Cip1^ knockdown enhances spheroid formation and invasion. **D**, **E** ACTL6A knockdown increases p21^Cip1^ protein level and reduces invasion, and these actions are partially reversed following simultaneous p21^Cip1^-siRNA and ACTL6A knockdown. **F**–**I** ACTL6A knockout lines display enhanced p21^Cip1^ levels and reduced spheroid formation. p21^Cip1^ knockdown in these lines increases spheroid formation, invasion, and migration. This suggests that p21^Cip1^ is a mediator of ACTL6A suppression of the cancer phenotype. Single asterisks indicate a significant decrease (*n* = 3, *p* ≤ 0.001) and double asterisks indicate a significant increase (*n* = 3, *p* ≤ 0.001).

The studies in Fig. 1 show that the p21^Cip1^ cyclin-dependent kinase inhibitor level is markedly increased in ACTL6A knockdown and knockout cells, suggesting that p21^Cip1^ may have a role in suppressing the cancer phenotype. To test this, we treated Meso-1 cells with control- and p21^Cip1^-siRNA and monitored the impact of p21^Cip1^ loss on the cancer phenotype. Figure 2A confirms p21^Cip1^ knockdown and shows that loss of p21^Cip1^ minimally impacts ACTL6A level. Figure 2B, C show that p21^Cip1^ knockdown enhances cancer cell aggression as evidenced by increased spheroid formation and invasion. To understand the relationship between ACTL6A and p21^Cip1^ level, and the cancer cell phenotype, we treated cells with combinations of ACTL6A- and p21^Cip1^-siRNA and monitored the impact on cell invasion. Figure 2D shows that ACTL6A knockdown increases p21^Cip1^ and Fig. 2E shows that this is associated with reduced invasion. Moreover, preventing the increase in p21^Cip1^ in ACTL6A knockdown cells (using p21^Cip1^-siRNA) (Fig. 2D) partially restores cell invasion (Fig. 2E), suggesting that the increase in p21^Cip1^ is responsible for suppression of the cancer phenotype in ACTL6A knockdown cells. We also studied the role of p21^Cip1^ using ACTL6A knockout cells. Figure 2F shows, as expected, that p21^Cip1^ level is elevated in ACTL6A knockout cells and that p21^Cip1^ is reduced by treatment with p21^Cip1^-siRNA. Figure 2G–I show that p21^Cip1^ knockdown in the ACTL6A knockout environment partially restores spheroid formation, invasion, and migration. These findings suggest that ACTL6A suppresses p21^Cip1^ to enhance the cancer phenotype.

**Fig. 3:**
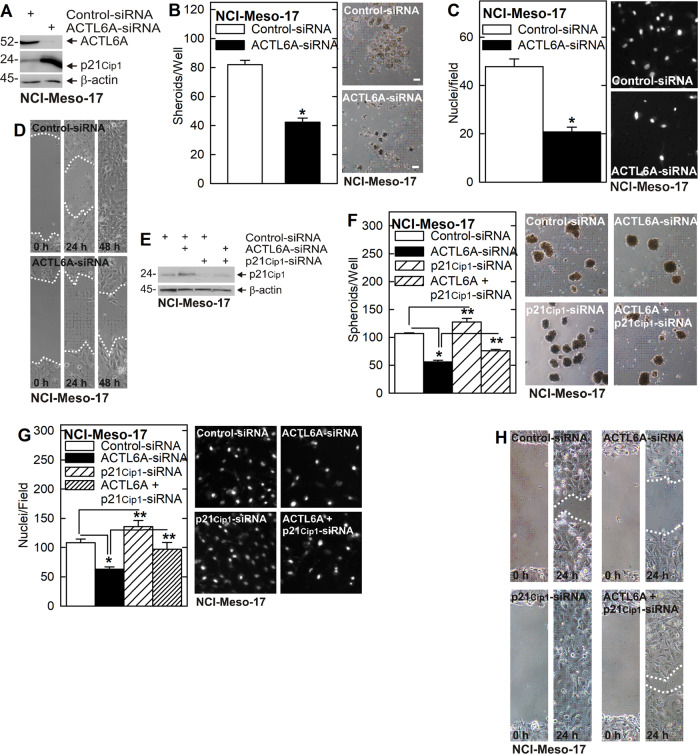
ACTL6A maintains the NCI-Meso-17 cancer cell phenotype. **A**–**D** ACTL6A knockdown increases p21^Cip1^ protein level and is associated with reduced spheroid formation, invasion, and migration. **E**–**H** ACTL6A knockdown increases p21^Cip1^ levels and reduces spheroid formation, invasion, and migration and p21^Cip1^ knockdown partially reverses the ACTL6A-knockdown dependent reduction in spheroid formation, invasion, and migration. These findings suggest that ACTL6A suppresses p21^Cip1^ to maintain the aggressive NCI-Meso-17 cell cancer phenotype. Single asterisks indicate a significant decrease (*n* = 3, *p* ≤ 0.001) and double asterisks indicate a significant increase (*n* = 3, *p* ≤ 0.005).

We next examined the role of ACTL6A and p21^Cip1^ in regulating the cancer phenotype in another mesothelioma cell line, NCI-Meso-17 cells. Figure 3A–D shows that ACTL6A knockdown increases p21^Cip1^ level and that this is associated with reduced NCI-Meso-17 cell spheroid formation, invasion, and migration. Moreover, as shown in Fig. 3E–H suppression of ACTL6A increases p21^Cip1^ and reduces the cancer phenotype, p21^Cip1^ knockdown enhances the cancer phenotype, and suppression of both ACTL6A and p21^Cip1^ produces an intermediate phenotype for spheroid formation, invasion, and migration.

### ACTL6A control of p21^Cip1^ expression

**Fig. 4:**
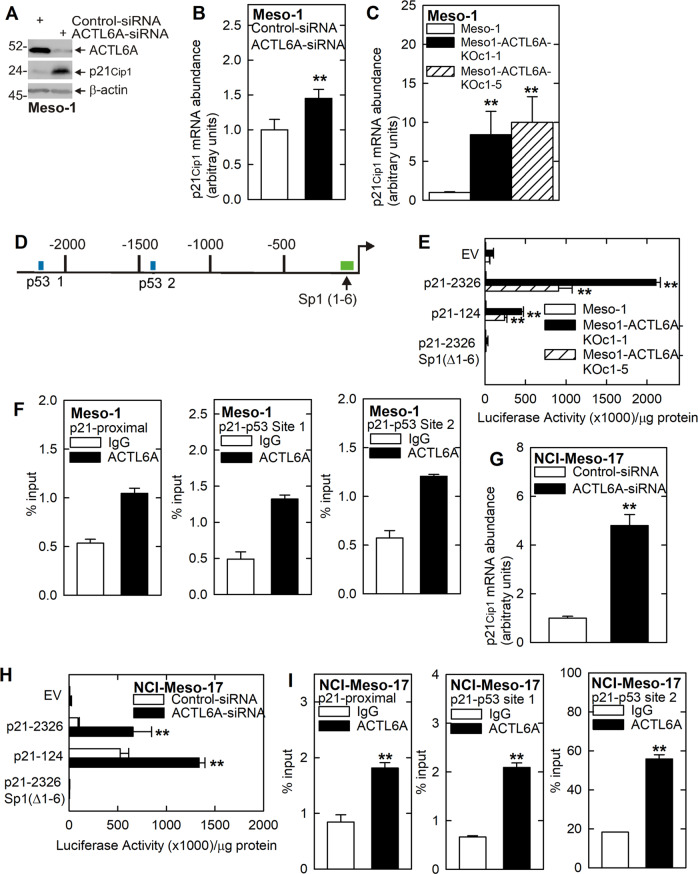
ACTL6A suppresses p21^Cip^ gene transcription to maintain the mesothelioma cancer phenotype. **A**, **B** ACTL6A knockdown increases Meso-1 cell p21^Cip1^ mRNA expression and protein level. **C** ACTL6A knockout Meso-1 cells express increased p21^Cip1^ mRNA. **D** Map of the full-length p21^Cip1^ promoter showing the p53-1 and p53-2 sites in the distal promoter and six Sp1 site in the proximal promoter. The top sequence indicates the authentic p53 site sequences. Our previous report indicates the mutations that inactivate these sites [28]. **E** ACTL6A knockout Meso-1 cells display enhanced p21^Cip1^ promoter activity for the full-length (p21-2326) and proximal (p21-124) promoter, and mutation of the six Sp1 sites eliminates the response. **F** Chromatin IP, using Meso-1 extracts, shows ACTL6A binding to the p21^Cip1^ promoter p53 binding sites and the proximal promoter Sp1 sites. **G**–**I** ACTL6A knockdown in NCI-Meso-17 cells increases p21^Cip1^ mRNA level and p21^Cip1^ promoter activity and this is associated with ATL6A binding to the p21^Cip1^ promoter p53 and Sp1 sites. As observed for Meso-1 cells, mutation of the proximal Sp1 sites eliminates transcriptional activity. Double asterisks indicate a significant increase (*n* = 3, *p* ≤ 0.001).

To understand the mechanism whereby ACTL6A regulates p21^Cip1^ expression, we compared p21^Cip1^ mRNA level in wild-type, ACTL6A knockdown and ACTL6A knockout Meso-1 cells. ACTL6A knockdown leads to an increase in p21^Cip1^ protein and mRNA (Fig. 4A, B), a finding that is consistent with evidence that restoring ACTL6A in ACTL6A knockout cells reduces p21^Cip1^ protein and mRNA (Fig. 1J). In addition, Fig. 4C shows that p21^Cip1^ mRNA level is markedly increased in ACTL6A knockout cells which is likely due to altered mRNA turnover or increased gene expression [28, 29].

We next examined the effect of ACTL6A on p21^Cip1^ transcription. The p21^Cip1^ promoter map (Fig. 4D) shows the six Sp1 sites located proximal to the transcription start site and the more distally located p53-1 and p53-2 sites [28, 29]. Activity of the p21-2326 (full-length) and p21-124 (proximal) promoter constructs are markedly increased in ACTL6A knockout cells suggesting activation of transcription (Fig. 4E). In contrast, the p21-2326 Sp1(Δ1-6) construct, in which the six Sp1 sites are mutated, is inactive, suggesting that the Sp1 elements are required for basal promoter activity and ACTL6A regulation of transcription. We next measured ACTL6A interaction at the proximal Sp1 sites, and the p53-1 and p53-2 sites because they constitute major p21^Cip1^ promoter response elements [22, 28, 30–32]. ChIP analysis shows that ACTL6A is enriched on these regulatory elements (Fig. 4F) suggesting that ACTL6A interacts at these sites to suppress p21^Cip1^ transcription.

We next examined ACTL6A regulation of p21^Cip1^ level in NCI-Meso-17 cells [33]. We show that ACTL6A knockdown increases p21^Cip1^ mRNA level (Fig. 4G) and promoter activity (Fig. 4H). In addition, the lack of activity of the p21-2326 Sp1(Δ1-6) construct (Fig. 4H), where all six Sp1 sites are inactivated, confirms that the promoter proximal Sp1 sites are required for basal and ACTL6A regulated promoter activity. ChIP analysis (Fig. 4I) shows that ACTL6A interacts at the p21^Cip1^ proximal Sp1 elements and the p53-1 and p53-2 sites. These findings strongly suggest that ACTL6A regulates p21^Cip1^ gene expression via a transcriptional mechanism in both Meso-1 and NCI-Meso-17 cells.

### Role of p53

**Fig. 5:**
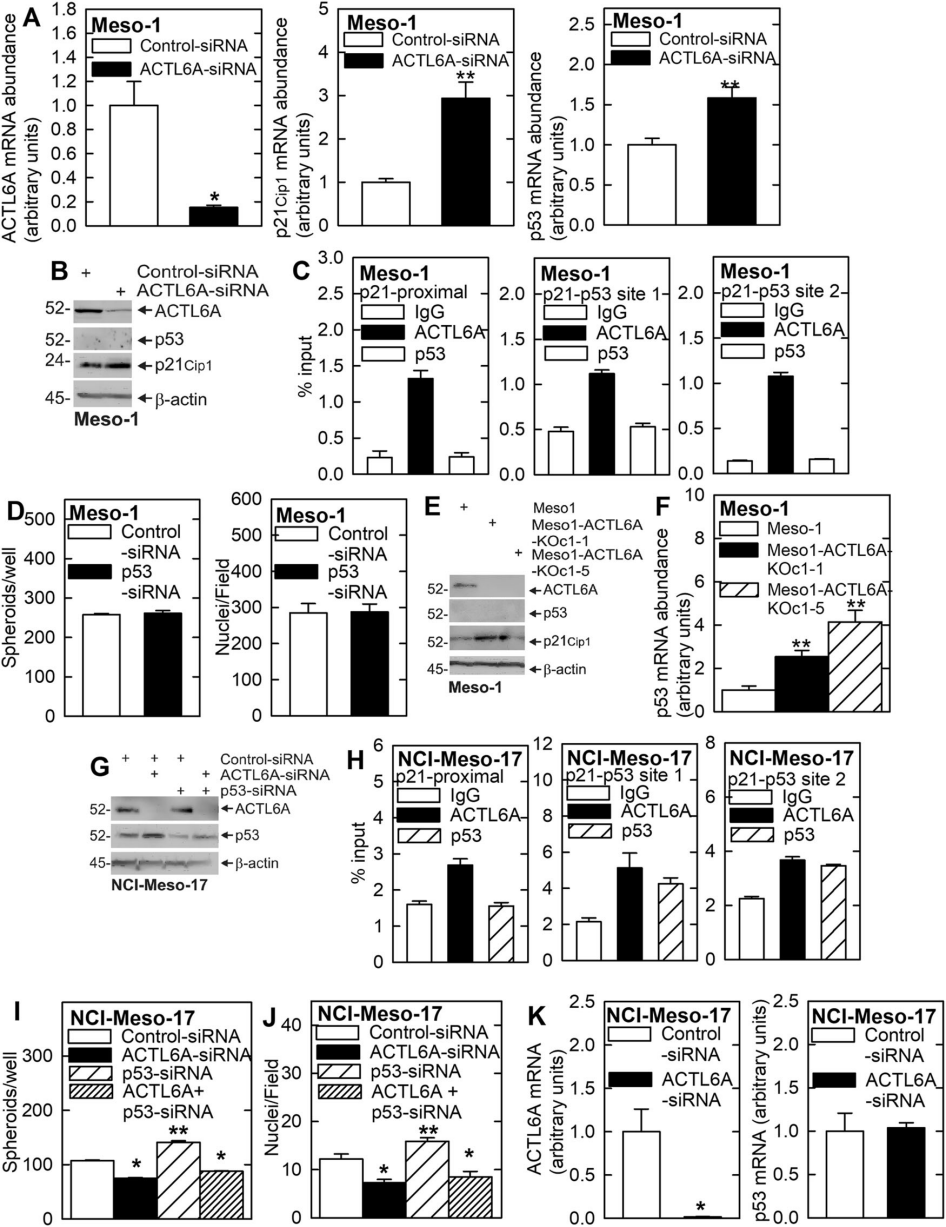
Role of p53 in mediating ACTL6A regulation of p21^Cip1^ expression. **A**, **B** ACTL6A-knockdown increases p53 and p21^Cip1^ mRNA levels and p21^Cip1^ protein level, but p53 protein is not detected in ACTL6A competent or knockdown Meso-1 cells. **C** Chromatin IP assay shows ACTL6A binding to the distal p53 binding sites and the proximal promoter Sp1 binding sites in Meso-1 cell extracts, but no binding of p53 is detected. **D** p53 knockdown does not alter Meso-1 cell spheroid number or invasion. **E**, **F** p53 protein is absent in ACTL6A knockout cells even though p53 mRNA levels are increased. **G** ACTL6A knockdown increases p53 in NCI-Meso-17 cells. **H** ACTL6A binds to the p21Cip1 promoter Sp1 and p53 sites. p53 binds to the p53 binding sites (p21-p53 sites 1 and 2) but not the Sp1 binding sites (p21-proximal). **I**, **J** ACTL6A knockdown reduces spheroid formation and invasion, p53 knockdown increases spheroid formation and invasion, and combination treatment with ACTL6A- and p53-siRNA does not alter ACTL6A suppression of spheroid formation and invasion. This suggests that ACTL6A suppresses the ability of p53 to activate p21^Cip1^ promoter transcription. **K** ACTL6A knockdown does not increase p53 mRNA level. Single asterisks indicate a significant decrease (*n* = 3, *p* ≤ 0.001) and double asterisks indicate a significant increase (*n* = 3, *p* ≤ 0.001). All statistical comparisons are to the control group. Single asterisks indicate a significant decrease (*n* = 3, *p* ≤ 0.001) and double asterisks indicate a significant increase (*n* = 3, *p* ≤ 0.001).

p53 is a well characterized regulator of p21^Cip1^ expression that binds at the p53 sites on the p21^Cip1^ promoter to increase gene expression [28, 30–32]. It is thus possible that ACTL6A loss leads to an increase in p53 which stimulates p21^Cip1^ transcription. We therefore examined the role of p53. Figure 5A shows that ACTL6A-siRNA reduces Meso-1 cell ACTL6A mRNA and that this is associated with an increase in p53 and p21^Cip1^ mRNA. Moreover, this is also associated with an increase in p21^Cip1^ protein (Fig. 5B). However, a surprising finding is that p53 protein is not detected in ACTL6A expressing or knockdown cells (Fig. 5B) which is consistent with the lack of p53 binding to the p21^Cip1^ promoter (Fig. 5C) and the fact that treatment with p53-siRNA does not alter Meso-1 spheroid formation or invasion (Fig. 5D). To provide additional evidence for the lack of p53 protein in Meso-1 cells, we confirmed an absence of p53 in ACTL6A knockout cells (Fig. 5E). It is interesting that p53 protein is absent even though p53 mRNA levels are increased in ACTL6A knockout cells (Fig. 5F). Taken together, these findings indicate that p53 is absent in Meso-1 cells and therefore has no role in regulating p21^Cip1^ gene expression.

We next examined the role of p53 as a regulator of p21^Cip1^ expression in NCI-Meso-17 cells. In contrast to Meso-1 cells, NCI-Meso-17 cells express p53, and p53 protein level is increased following ACTL6A knockdown (Fig. 5G). Moreover, treatment with p53-siRNA attenuates the p53 increase in ACTL6A knockdown cells (Fig. 5G). This suggests that p53 may have a role in mediating the increase in p21^Cip1^ following ACTL6A knockdown. If p53 has a role in this induction, we would anticipate that it should interact with the p21^Cip1^ promoter. In Fig. 5H we confirm that both ACTL6A and p53 interact with the p21^Cip1^ promoter p53 binding sites, but that only ACTL6A interacts at the proximal promoter Sp1 sites. We next examine the impact of ACTL6A, p53 and combined knockdown on NCI-Meso-17 cell spheroid formation and invasion. Figure 5I, J show that ACTL6A knockdown reduces and p53 knockdown increases spheroid formation and invasion, and that combined ACTL6A and p53 knockdown reduces spheroid formation and invasion. We also examined the impact of ACTL6A knockdown on p53 mRNA level in NCI-Meso-17 cells and found no impact (Fig. 5K). Taken together, these findings are consistent with a role for p53 in mediating ACTL6A suppression of p21^Cip1^ levels in NCI-Meso-17. This suggests two potential mechanisms of ACTL6A action. First, the loss of ACTL6A increases p53 protein level (via stabilization or enhanced translation) which stimulates p21^Cip1^ gene expression. Second, reduced ACTL6A binding to the p53 sites on the p21^Cip1^ promoter, following ACTL6A knockdown, likely permits p53 to drive p21^Cip1^ promoter activity in an unimpeded manner.

### ACTL6A is required for optimal tumor growth

**Fig. 6:**
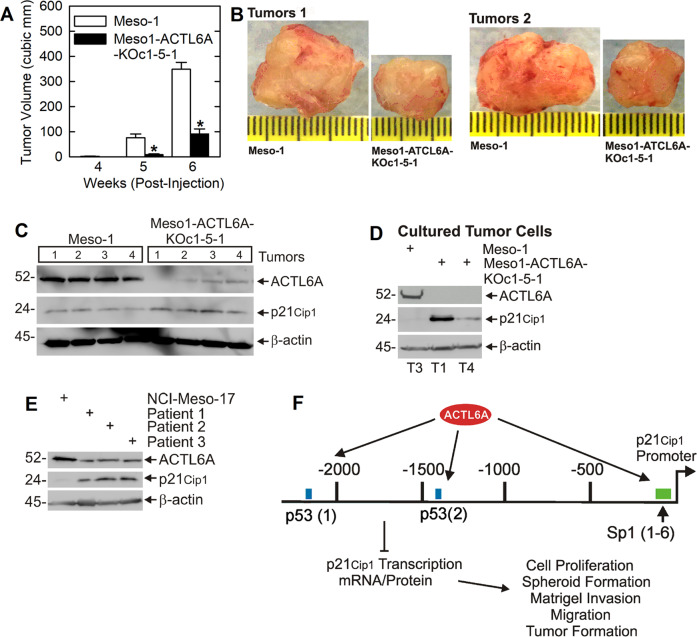
ACTL6A suppresses p21^Cip1^ level to maintain tumor growth. **A**, **B** Wild-type (Meso-1) and ACTL6A knockout (Meso1-ACTL6A-KOc1-5-1) cells (3 million/site) were injected into each front flank in five NSG mice per treatment group (10 tumor/group) and tumor formation was monitored for 0–6 week. The images are two paired sets of tumors harvested at 6 weeks. Differences in tumor formation were analyzed using the Student’s *t* test and values are presented as mean ± SEM and *p* values are indicated. Single asterisks indicate a significant decrease (*n* = 10 tumor, *p* ≤ 0.001). **C** Meso-1 cell tumors display low levels of p21^Cip1^ while p21^Cip1^ levels are elevated in Meso1-ACTL6A-KOc1-5-1 cell tumors. **D** Meso-1 and Meso1-ACTL6A-KOc1-5-1 tumors cells were dissociated and grown in culture for detection of ACTL6A and p21^Cip1^. These blots show that the Meso1-ACTL6A-KOc1-5-1 cells retained their ACTL6A-null status during tumor formation. **E** ACTL6A and p21^Cip1^ levels were compared between NCI-Meso-17 cells and tumors isolated from three patients. **F** Schematic describing the role of ACTL6A in regulating cancer cell function. ACTL6A binds to the p21^Cip1^ promoter p53-1 and 2 sites and the Sp1 sites to suppress p21^Cip1^ transcription. The resulting reduction in p21^Cip1^ level leads to enhanced cell proliferation, spheroid formation, Matrigel invasion, migration, and tumor formation.

To assess the role of ACTL6A in tumor formation, we examined the ability of monolayer cultured wild-type and ACTL6A null cells to form tumors in immune-compromised mice. Figure 6A, B show that ACTL6A knockout cells (Meso1-ACTL6A-KOc1-5-1) display reduced tumor growth. Figure 6C shows that loss of ACTL6A in tumors is associated with increased p21^Cip1^ expression, a result that is consistent with the findings from cultured cells. However, we were surprised to detect residual low level ACTL6A expression in Meso1-ACTL6A-KOc1-5-1 cells. To confirm that ACTL6A knockout status of these cells, we harvested cells from Meso-1 and Meso1-ACTL6A-KOc1-5-1 tumors and grew them in cell culture for 2 weeks prior to extract preparation for ACTL6A immunoblot. As shown in Fig. 6D, no ACTL6A was detected in the cultured Meso1-ACTL6A-KOc1-5-1 cells. This confirms that Meso1-ACTL6A-KOc1-5-1 cells retained the ACTL6A-null phenotype and that the ACTL6A detected in Fig. 6C is derived from connective tissue cells in the tumor.

### ACTL6A and p21^Cip1^ in patient tumor samples

We next checked ACTL6A and p21^Cip1^ levels in patient tumors. Figure 6E compares ACTL6A and p21^Cip1^ levels in NCI-Meso-17 cells with tumor samples derived from three pleural mesothelioma patients. The highest level of ACTL6A is observed in NCI-Meso-17 cells which are negative for p21^Cip1^ expression. In contrast, ACTL6A levels are lower in the patient samples and this is associated with basal levels of p21^Cip1^ expression. Based on our model, where high ACTL6A expression reduces p21^Cip1^, we interpret these results as suggesting that the tumor samples express enough ACTL6A to partially, but not completely suppress, p21^Cip1^ expression.

## Discussion

### ACTL6A enhances the mesothelioma cancer phenotype

Mesothelioma is an aggressive and incurable disease that is associated with poor life expectancy [1, 2]. This disease profile has stimulated efforts to identify new treatment targets. ACTL6A has been reported to drive cancer progression by stabilizing the YAP1/TAZ pro-cancer transcriptional regulators [18, 19], and by suppressing expression of the p21^Cip1^ cyclin-dependent kinase inhibitor [20–23]. Moreover, ACTL6A maintains the stem-like phenotype in keratinocytes [13], stimulates neural progenitor cell proliferation [34, 35], enhances the osteosarcoma and hepatocellular carcinoma cell cancer phenotype [16, 17], and drives an aggressive epidermal squamous cell carcinoma phenotype [22]. To our knowledge ACTL6A function has not been studied in mesothelioma; however, it is important to examine the role of ACTL6A in mesothelioma, since it regulates key pathways, including YAP1/TAZ/TEAD and p21^Cip1^ signaling, that have been implicated as important in mesothelioma cancer progression [24–27].

We show that transient ACTL6A knockdown or stable knockout reduces mesothelioma cell proliferation, spheroid formation, invasion, and migration. This suggests that ACTL6A maintains an aggressive mesothelioma cancer phenotype. To understand the mechanism of action, we show that p21^Cip1^ level is suppressed in ACTL6A competent cells and is increased in ACTL6A knockout cells, suggesting that ACTL6A may maintain the cancer phenotype by suppressing p21^Cip1^ expression. A functional role for p21^Cip1^ was confirmed by showing that suppressing p21^Cip1^ level in ACTL6A knockdown or knockout cells, attenuates the anti-cancer response associated with ACTL6A loss. A key role of p21^Cip1^ was confirmed by showing that p21^Cip1^ knockdown results in increased cancer cell invasion and migration thereby confirming that the increase in p21^Cip1^ in ACTL6A negative cells suppresses the cancer phenotype. These findings strongly suggest that p21^Cip1^ is a biologically important downstream mediator of ACTL6A action.

### ACTL6A regulation of p21^Cip1^ level

Sp1 and p53 transcription factors are key regulators of p21^Cip1^ gene expression that bind to key DNA elements on the p21^Cip1^ promoter to increase expression [30, 36]. We therefore examined ACTL6A interaction at these sites. Mechanistic studies show that in Meso-1 cells ACTL6A binds to the p21^Cip1^ gene Sp1 site-rich proximal promoter and the distal p53-1 and 53-2 sites, and that this is associated with reduced p21^Cip1^ mRNA and protein levels. This is consistent with a role for ACTL6A as a dominant suppressor of p21^Cip1^ expression. Since, p53 is an important factor that binds to the p21^Cip1^ promoter to activate transcription, we assessed its role. Our findings show that ACTL6A loss in Meso-1 cells is associated with increased p53 mRNA, but that p53 protein is not produced and is not found bound to the p21^Cip1^ promoter p53 binding sites. Thus, p53 does not have a p21^Cip1^ regulatory role in Meso-1 cells. This finding in Meso-1 cells is consistent with a previous report showing p53 is not required for ACTL6A regulation of p21^Cip1^ in epidermal squamous cell carcinoma cells [22]. Taken together, these finding suggest that in Meso-1 cells ACTL6A interaction at these sites suppresses ability of other, presently unknown, transcription factors to activate p21^Cip1^ expression.

ACTL6A was also found to suppress p21^Cip1^ expression in NCI-Meso-17 cells where it is bound to the p21^Cip1^ gene promoter at Sp1 and the p53 binding sites where it likely acts to suppress p21^Cip1^ expression. Moreover, ACTL6A knockdown resulted in an increase in p21^Cip1^ and an attenuated cancer phenotype. These findings suggest that ACTL6A binds to the Sp1 and p53 binding sites to reduce the ability of other transcription factors to activate expression. However, in contrast to Meso-1 cells, we found that NCI-Meso-17 cells do express p53 protein. We show that although p53 mRNA levels are not increased in NCI-Meso-17 ACTL6A knockdown cells, p53 protein level is increased. p53 and ACTL6A were both found to be able to bind at the p21^Cip1^ promoter p53 binding sites. Taken together, these findings suggest that ACTL6A suppresses p53 protein level. In addition, ACTL6A binds to the p21^Cip1^ promoter p53 binding site where it likely suppresses p53-dependent activation of p21^Cip1^ transcription. This is consistent with the previous evidence that ACTL6A can interfere with p53 function [23]. Additional studies will be required to fully understand the relationship between ACTL6A, p53 and p21^Cip1^ in NCI-Meso-17 cells.

### The role of the p21^Cip1^ proximal promoter Sp1 sites

We have previously identified an important role for the cluster of six Sp1 sites located in the proximal p21^Cip1^ promoter [28]. The present studies indicate that these sites must be intact for p21^Cip1^ basal transcription since mutation of the Sp1 sites eliminates promoter activity. Thus, these sites are required for basal promoter activity and are also likely important for ACTL6A suppression of transcription, as chromatin IP analysis reveals ACTL6A binding at these sites in both Meso-1 and NCI-Meso-17 cells. Although it appears that ACTL6A binding to these sites may inhibit Sp1 activation of p21^Cip1^ transcription, additional studies will be necessary for a detailed description of the mechanism of action. It is important to note that we have focused our studies on major regulatory sites (p53-1, p53-2, and the Sp1 cluster) that are known to drive p21^Cip1^ transcription. However, additional studies will ultimately be necessary, as it is very likely that ACTL6A can interact with other transcriptional regulators that bind to other regions of the p21^Cip1^ promoter and that these interactions will influence p21^Cip1^ levels.

### ACTL6A regulation of tumor formation

To assess the biological relevance of these findings, we tested the impact of ACTL6A knockout on tumor formation. Wild-type Meso-1 cells and ACTL6A knockout cells (Meso1-ACtL6A-KOc1-5-1) cells were injected into immunocompromised mice to monitor the impact of ACTL6A loss on tumor formation. We observe a dramatic reduction in tumor size for ACTL6A knockout tumors. Biochemical analysis of tumor extracts confirms ACTL6A knockout and shows that this is associated with increased levels of p21^Cip1^. Thus, ACTL6A loss results in increased p21^Cip1^ levels and this is associated with an attenuated cancer phenotype. The fact that ACTL6A knockout leads to increased p21^Cip1^ levels and reduced tumor growth argues that ACTL6A suppresses p21^Cip1^ to maintain aggressive tumor growth. These findings are completely consistent with the experiments in cultured cells and show that ACTL6A operates in the tumor context to suppress p21^Cip1^ level to drive an aggressive cancer phenotype.

Based on these findings we propose the model shown in Fig. 6F where ACTL6A interacts at the p53 and Sp1 transcription factor binding sites to interfere with positive transcriptional regulators (such as p53) to reduce p21^Cip1^ promoter expression and enhance the cancer phenotype.

## Materials and methods

### Reagents

ChIP grade Rabbit anti-ACTL6A (A301-391A) was obtained from Bethyl Laboratories (Montgomery, TX). ChIP grade rabbit anti-p53 (9282), ChIP grade anti-IgG (2792), and anti-p21^Cip1^ (2947) were obtained from Cell Signaling Technologies (Danvers, MA). Secondary rabbit anti-IgG for immunoprecipitation (NI01), anti-β-actin (A5441), and anti-FLAG (E3165) were purchased from Millipore/Sigma (St. Louis, MO). Control (sc-37007), p53 (sc-44218) and p21^Cip1^ (sc-29427) siRNA were obtained from Santa Cruz Biotechnology (Dallas, TX). ACTL6A-siRNA (AM16708) was obtained from Ambion (Philadelphia, PA). The pCMV3-ACTL6A-FLAG expression vector (HG10963-CF) was obtained from Sino Biologicals (Wayne State, PA).

### Statistics

The two-tailed Student’s *t* test was used for statistical assessment. The values in plots are mean ± SEM. Single and double asterisks indicate, respectively, a significant reduction and increase in response using the Student’s *t* test based on triplicates for cell cultures studies and 5 mice/group for animal tumor growth assays. No data was excluded from analysis.

### Cell culture and bioassays

Meso-1 and NCI-Meso-17 cells are derived, respectively, from peritoneal and pleural mesothelioma [5, 33] and mycoplasma testing is performed when new cell stocks are thawed for use. RPMI1640 medium (27519003) containing l-glutamine, penicillin-streptomycin solution, and 0.25% trypsin-EDTA were purchased from Gibco (Gaithersburg, MD). Growth medium is RPMI1640 supplemented with penicillin–streptomycin and 5% fetal bovine serum (FBS, 19B416) which was purchased from Sigma (St. Louis, MO). To study cell growth, 20,000 cells were plated into 35 mm plates in triplicate in growth medium and cell number was monitored from 0–2 days. For spheroid formation assay, cells were plated at 40,000 cells per 35 mm well in ultra-low attachment dishes [37, 38]. To study invasion, 25,000 cells were plated into Millicell (1 cm diameter, 8 mm pore-size) chambers (353097) atop a 100 μl layer of 250 μg/ml matrigel (354234) [39]. Millicell chambers and matrigel were purchased from BD Biosciences (San Diego, CA). Growth medium containing 1% FCS (top chamber) or 10% FCS (bottom chamber) was added and the ability of cells to pass through the membrane was monitored over 0–18 h. The membrane was fixed with 4% paraformaldehyde and stained with DAPI (D9542, Sigma Aldrich, Milwaukee, WI) and fluorescent nuclei were detected by fluorescence microscopy [39]. For migration assay, scratch wounds were created in confluent monolayer cultures of cells using a pipette tip and closure of the wound was monitored from 0 to 48 h.

### Electroporation

Cells (1.2 million), suspended in 100 μl of nucleofection reagent (VPD-1002, Lonza, Williamsport, PA) containing 3 μg of control- or target specific-siRNA, were electroporated using the AMAXA Electroporator on the T-018 setting [40]. The cells were permitted to recover for 48 h and then electroporation was repeated. After a 12 h recovery, the cells were plated to assay spheroid formation, invasion, and migration [37, 38]. For ACTL6A-FLAG expression studies, cells were single electroporated with 1 µg of pCMV3-ACTL6A-FLAG expression vector and ACTL6A-FLAG expression and biological responses were monitored after 48 h.

### p21^Cip1^ Promoter assay

To measure the impact of ACTL6A on p21^Cip1^ promoter activity, cells were double-electroporated with 3 μg of control- or ACTL6A-siRNA and plated onto duplicate 12-well plates. After overnight attachment, 1 μg of reporter plasmid was mixed with 3 μl of Fugene-6 for transfection and at 18–24 h post-transfection extracts were assayed for luciferase activity assay [28]. The p21^Cip1^ promoter reporter constructs, p21-2326 and p21-124 were cloned in empty pBluescript-Luc vector (EV). p21-124 encodes the proximal promoter region. p21-2326 encodes the intact full-length p21^Cip1^ promoter fused to luciferase and p21-2326 Sp1(Δ1-6) is the full-length promoter in which the six proximal Sp1 sites are mutated [28, 36].

### Immunology related methods

Immunoblot was performed as previously described [28]. Chromatin immunoprecipitation (ChIP) was performed as described in the Diagenode LowCell# ChIP kit (kch-maglow-G48, Diagenode, Inc., Denville, NJ) protocol. Cells (1 million) were harvested and washed with phosphate buffered saline. Sodium butyrate was omitted from the protocol. The cells were crosslinked with 1% formaldehyde for 10 min, quenching with 125 nM glycine and washed with phosphate-buffered saline before lysis in Diagenode lysis buffer supplemented with 1x protease inhibitor. The samples were chilled and sonicated using a 550 Sonic Dismembrator (five 30 s pulses at 30% amplitude with 30 s between pulses) to obtain 200–1000 bp DNA fragments of sheered DNA. The samples were diluted in Diagenode buffer containing 1x protease inhibitor. ChIP grade antibodies (1 μg) were incubated with protein G coated magnetic beads for 3 h at 4 °C, and sheared chromatin (100,000 cells equivalent) was added for overnight 4 °C incubation. After 24 h, the DNA was extracted using Diagenode DNA isolation buffer containing proteinase K. p21^Cip1^ promoter DNA were detected by qPCR using LightCycler 480 SYBR green I master mix and sequence specific primers. p21^Cip1^ promoter primers targeted the proximal Sp1 elements (forward 5′-GCTGGGCAGCCAGGAGCCTG and reverse 5′-CTGCTCACACCTCAGCTGGC), the p53-1 site (forward 5′-GTGGCTCTGATTGGCTTTCTG and reverse 5′-CTGAAAACAGGCAGCCCAAG) and the p53-2 site (forward 5′-CCGAGGTCAGCTGCGTTAGAGG and reverse 5′-AGAACCCAGGCTTGGAGCAGC).

### ACTL6A knockout cells

ACTL6A-specific CRISPR guide RNA, forward (5′-caccGGCGATAAAGGCAAACAAGG) and reverse (5′-aaacCCTTGTTTGCCTTTATCGCC), were identified using tools at http://crispr.technology and cloned into the U6-driven pSpCas9(BB)-2A-Puro (PX459) V2.0 vector from Addgene. The vector (3 μg) was electroporated into Meso-1 cells using the AMAXA electroporator and at 48 h post-electroporation the cells were treated with 2 μg/ml puromycin for 24 h followed by selection of single cell clones by dilution cloning in drug-free medium. The resulting clonal ACTL6A knockout lines include Meso1-ACTL6A-KOc1-1 and Meso1-ACTL6A-KOc1-5. Meso1-ACTL6A-KOc1-5 cells were subjected to an additional round of clonal selection to create Meso1-ACTL6A-KOc1-5-1 cells which were used in tumor experiments.

### qRT-PCR

RNA was isolated using Illustra RNAspin Mini kit (25050070, GE Healthcare, Chicago, IL), reverse-transcribed and quantified using the LightCycler 480 PCR system (Roche Life Science, Branford, CT). Target specific PCR primers were used to quantify the transcript level and signals were normalized to cyclophilin A. Primers included p21^Cip1^ forward (5′-CGTCTGCAACCACAGGGATTTCTT-3′) and reverse (5′-TGTTGATTGTCACATGCTTCCGGG-3′), p53 forward (5′-TAACAGTTCCTGCATGGGCGGC-3′) and reverse (5′-AGGACAGGCACAAACACGCACC-3′), and ACTL6A forward (5′-TGGAGGCCATTTCACCTCTAA-3′) and reverse (5′-TCTTTGCTCTAGTATTCCACGGT’).

### Tumor xenografts

Cancer cells (3 million), derived from monolayer cultures, were re-suspended in 200 µl of phosphate-buffered saline containing 30% matrigel and 100 µl was injected subcutaneously into each front flank of five 8-week-old NOD/scid/IL2 receptor γ chain knockout mice (NSG) per treatment group. Tumor growth was monitored by measuring tumor diameter and calculating tumor volume = 4/3π × (diameter/2)3. Tumor samples were harvested to prepare extracts for immunoblot [4]. The two-tailed Student’s *t* test was used for statistical analysis. Power analysis, assuming a power of 0.8, standard deviation of 12%, and a minimum difference in means of 30%, predicts that five animals per group (2 tumors/mouse = 10 tumors) are sufficient to provide statistically relevant outcomes. Human pleural mesothelioma tumor samples were obtained from patients as discarded tissue samples.
